# U-shaped association between serum Klotho and accelerated aging among the middle-aged and elderly US population: a cross-sectional study

**DOI:** 10.1186/s12877-023-04479-9

**Published:** 2023-11-28

**Authors:** Heng Li, Shuai Miao, Min Zhang, Peng Zhang, Yan-Bin Li, Rui-Sheng Duan

**Affiliations:** 1 Department of Neurology, The First Affiliated Hospital of Shandong First Medical University & Shandong Provincial Qianfoshan Hospital, Jinan, 250014 People’s Republic of China; 2 Shandong Institute of Neuroimmunology, Jinan, 250014 People’s Republic of China; 3 Medical School of Chinese People’s Liberation Army (PLA), Beijing, 100853 People’s Republic of China; 4 Department of Neurology, the First Medical Center, Chinese PLA General Hospital, Beijing, 100853 People’s Republic of China

**Keywords:** Klotho, Phenotypic age, Accelerated aging, U-shaped, NHANES

## Abstract

**Background:**

Phenotypic age acceleration, which reflects the difference between phenotypic age and chronological age, is an assessment to measure accelerated aging. Klotho is a protein related to slower aging, but its association with accelerated aging remains unclear.

**Methods:**

Based on data from the 2007–2010 National Health and Nutrition Examination Survey, phenotypic age was calculated using chronological age and 9 aging-related biomarkers. A total of 4388 participants aged 40 to 79 years with measured serum Klotho and calculated phenotypic age were enrolled. The association between serum Klotho and phenotypic age acceleration was estimated using multivariable linear regression models. The possible nonlinear relationship was examined with smooth curve fitting. We also conducted a segmented regression model to examine the threshold effect.

**Results:**

The association between serum Klotho and phenotypic age acceleration followed a U-shaped curve (*p* for nonlinearity < 0.001), with the inflection point at 870.7 pg/ml. The phenotypic age acceleration significantly decreased with the increment of serum Klotho (per SD increment: β -1.77; 95% CI, -2.57 ~ -0.98) in participants with serum Klotho < 870.7 pg/ml, and increased with the increment of serum Klotho (per SD increment:β, 1.03; 95% CI: 0.53 ~ 1.54) in participants with serum Klotho ≥ 870.7 pg/ml.

**Conclusion:**

There was a U-shaped association between serum Klotho and accelerated aging among the middle-aged and elderly US population.

## Introduction

Populations around the world are aging at an unprecedented rate than those in the past. An extension of life is usually fraught with chronic disease and fragility, representing a challenge to health care systems [1]. However, health and function manifest differently in individuals with the same chronological age, suggesting that individuals differ in their rates of aging. Recently, phenotypic age, a new biological aging measure based on commonly used clinical biomarkers, has been proposed to capture morbidity and mortality risk in different subpopulations in US cohort studies [2]. Phenotypic age acceleration reflects the deviation between phenotypic and chronological age and indicates accelerated aging [3, 4]. Measures of accelerated aging by phenotypic age acceleration outperform chronological age in predicting aging outcomes [5].

Klotho was originally proposed to have anti-aging properties in 1997, when Kuro-o et al. reported that Klotho-deficient mice exhibit a short lifespan and multiple aging-related phenotypes, such as arteriosclerosis, hypoglycemia, osteoporosis, emphysema, skin atrophy and gonadal dysplasia [6]. Klotho has been reported to be associated with a variety of aging-related diseases, including chronic kidney disease, metabolic syndrome, cognitive decline and atherosclerosis [7–11]. Zhu et al. discovered that the Klotho gene polymorphisms are closely linked to healthy aging and longevity [12]. Secreted Klotho is emerging as a biomarker for predicting health status in the elderly [13]. However, the association between serum Klotho and accelerated aging has not been described. Therefore, we initially hypothesized that the serum Klotho might also be correlated with accelerated aging and aimed to explore the association in the middle-aged and elderly US population.

## Methods

### Study design and participants

National Health and Nutrition Examination Survey (NHANES) is a cross-sectional and nationally representative survey that collects a representative sample of around 5,000 people per year, aiming at estimating the health and nutritional status in the United States. The survey has been approved by the National Center for Health Statistics of the Centers for Disease Control and Prevention and all participants provided written informed consent for the study.

**Fig. 1:**
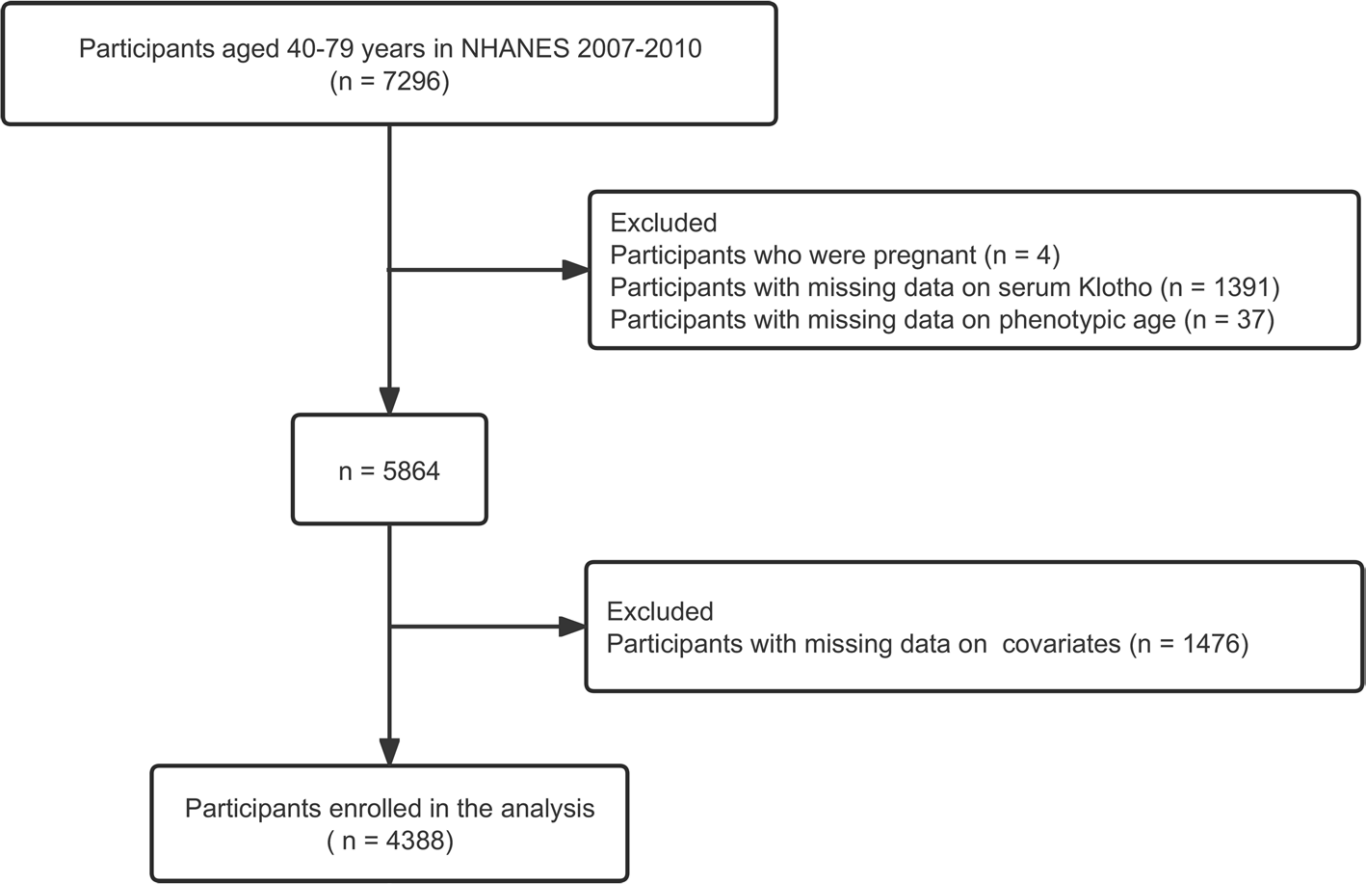
Flowchart of participant selection

Considering the availability of both Klotho and C-reactive protein (CRP) in serum, data were collected from NHANES 2007–2010, while serum CRP is an important biomarker to evaluate phenotypic age acceleration. There were 7296 participants aged 40 to 79 years between 2007 and 2010, and we initially excluded those who were pregnant or with missing data on serum Klotho or with incomplete data to calculate phenotypic age acceleration. Subsequently, we excluded those with missing data on important covariates, including smoking, sleep, hypertension, drinking, body mass index (BMI), education, marriage, and energy intake, leaving 4388 subjects eligible for further analyses (Fig. 1).

### Measurement of serum Klotho

Levels of serum Klotho were detected using an enzyme-linked immunosorbent assay (ImmunoBiological Laboratories, Gunma, Japan) according to the manufacturer’s instructions. Duplicate samples were detected and the average was calculated as the final result.

### Assessment of phenotypic age acceleration

Phenotypic age was calculated by using chronological age and 9 aging-related biomarkers, including albumin, creatinine, glucose, CRP, lymphocyte percent, mean cell volume, red blood cell distribution width, alkaline phosphatase, and white blood cell count [2]. Phenotypic age acceleration was the residual resulting from a linear model when regressing phenotypic age on chronological age.

### Measurement and definitions of covariates

Sociodemographic characteristics (age, sex, race, education, marriage, and family income), health condition (diabetes and hypertension) and information of other health behaviors (smoking, drinking, sleep duration and physical activity), were obtained from self-reported questionnaires. Race was categorized into Mexican Americans, other Hispanic, non-Hispanic white, non-Hispanic black and other races. Based on the educational level, participants were categorized into three groups, including not graduate from high school, high school graduation, and college education or above. Marriage status was categorized as married/living with partner, widowed/divorced/separated and never married. We used the poverty income ratio (PIR), calculated by the family size-specific threshold, to define family income (< 1.3, 1.3–3.5 and ≥ 3.5). The health condition data were composed of hypertension and diabetes history. Participants who met one of the following conditions were considered to have diabetes: (1) people who had been informed of diabetes by doctors or other health professionals; (2) people who were taking insulin; (3) people who were taking diabetic pills. Participants were considered to have hypertension if they met one of the followings: (1) they had been informed of hypertension by doctors or other health professionals; (2) they were taking a prescription for hypertension. Smoking status was classified as never (those who reported smoking fewer than 100 cigarettes in their lifetime), former (reported ever smoking at least 100 cigarettes in their lifetime but do not currently smoke), or current (smoked at least 100 cigarettes and currently smoke some days or every day). Drinking status was defined as yes if the participants had at least 12 alcohol drinks 1 year. Physical activity was calculated according to the metabolic equivalent (MET). A MET value for each activity was assigned according to the suggested MET scores by NHANES. A total activity score was calculated by summing the MET-hours of each activity in a week. Dietary intake was estimated by two 24-h dietary recall interviews. The first recall interview was collected in an NHANES Mobile Examination Center, and the second dietary recall interview was conducted by telephone after 3–10 days. The average energy intake of the two 24-h recalls was used for analysis. BMI was calculated using the participant’s weight in kilograms divided by height in meters squared (kg/m^2^) and categorized by 25 and 30 kg/m^2^.

### Statistical analysis

Participants were grouped into 5 categories based on quintiles of the serum Klotho concentrations. The baseline characteristics were summarized as mean ± standard deviations (SD) or median with interquartile range (IQR) for continuous variables and numbers (n) with percentages (%) for categorical variables. Tests of differences in characteristics across Klotho categories were performed using one-way ANOVA or the Kruskal–Wallis test for continuous variables and χ^2^ test for categorical variables.

A multiple linear regression model was applied to evaluate the association between serum Klotho levels and phenotypic age acceleration. Serum Klotho was entered as a categorical variable (quintiles) and as a continuous variable (with the β value calculated per SD increment). Four models were constructed. In the crude model, no confounders have been adjusted; in model 1, we adjusted for covariables that change the effect value by at least 10%; in model 2, covariables were further chosen when in univariable analysis, the P value was less than 0.1; and the covariables related to phenotypic age acceleration reported by previous research were further adjusted in model 3. We further applied a smoothing curve and a two-piecewise linear regression model to examine the possible nonlinear association between serum Klotho and phenotypic age acceleration. A likelihood ratio test was conducted to compare the one-line linear regression model with the two-piecewise linear model.

To assess possible effect modification, stratified analyses were performed according to age, sex, smoking, drinking, hypertension and diabetes. Interaction across subgroups was tested using the likelihood ratio test.

We also performed sensitivity analyses using multiple imputation, based on 5 replications and a chained equation approach method in the R package MICE.

Overall, all statistical analyses were performed using R (ver 4.2.1), SPSS (ver 26) and Free Statistics (ver 1.5). Sample weights were not adjusted in the present study. A two-sided value of *p* < 0.05 was considered statistically significant.

## Results

### Participant characteristics

Table 1 shows the population characteristics grouped by serum Klotho quintiles. Overall, 4388 participants were included, with a median age of 58 years, and 48.9% were male. The median serum Klotho concentration was 791.3 (648.3, 984.0) pg/ml. The median phenotypic age was 54.0 (43.9, 64.8) years. The median phenotypic age acceleration was -1.9 (-5.7, 2.7) years. Participants with higher levels of Klotho tended to be younger, female, and non-Hispanic black, less likely to be current smokers or drinkers.

**Table 1:** Baseline characteristics of participants by quintiles of serum Klotho

Variables		Serum Klotho (pg/mL)					*P* value
	Total	Quintile 1	Quintile 2	Quintile 3	Quintile 4	Quintile 5	
		(152.5–611.9)	(612.4–733.9)	(734.0–857.1)	(857.2–1048.4)	(1048.8–3829.7)	
Number	4388	878	876	879	877	878	
Age, year	58.1 ± 11.0	59.3 ± 11.2	58.3 ± 11.0	58.1 ± 11.0	57.9 ± 10.9	56.9 ± 10.7	< 0.001
Male, n (%)	2147 (48.9)	469 (53.4)	462 (52.7)	433 (49.3)	415 (47.3)	368 (41.9)	< 0.001
**Race, n (%)**							< 0.001
Mexican American	714 (16.3)	155 (17.7)	140 (16)	139 (15.8)	140 (16)	140 (15.9)	
Other Hispanic	422 ( 9.6)	76 (8.7)	70 (8)	90 (10.2)	92 (10.5)	94 (10.7)	
Non-Hispanic White	2344 (53.4)	466 (53.1)	487 (55.6)	513 (58.4)	469 (53.5)	409 (46.6)	
Non-Hispanic Black	774 (17.6)	152 (17.3)	155 (17.7)	116 (13.2)	136 (15.5)	215 (24.5)	
Other Race	134 ( 3.1)	29 (3.3)	24 (2.7)	21 (2.4)	40 (4.6)	20 (2.3)	
**BMI, n (%)**							0.965
< 25 kg/m^2^	998 (22.7)	200 (22.8)	190 (21.7)	193 (22)	202 (23)	213 (24.3)	
25–30 kg/m^2^	1561 (35.6)	310 (35.3)	317 (36.2)	316 (35.9)	306 (34.9)	312 (35.5)	
≥ 30 kg/m^2^	1829 (41.7)	368 (41.9)	369 (42.1)	370 (42.1)	369 (42.1)	353 (40.2)	
**Marriage, n (%)**							0.196
Married/Living with partner	2919 (66.5)	573 (65.3)	588 (67.1)	601 (68.4)	588 (67)	569 (64.8)	
Widowed/Divorced/Separated	1176 (26.8)	248 (28.2)	219 (25)	228 (25.9)	242 (27.6)	239 (27.2)	
Never married	293 ( 6.7)	57 (6.5)	69 (7.9)	50 (5.7)	47 (5.4)	70 (8)	
**Education, n (%)**							0.307
Did not graduate from high school	1280 (29.2)	250 (28.5)	275 (31.4)	238 (27.1)	244 (27.8)	273 (31.1)	
Graduated from high school	1023 (23.3)	215 (24.5)	197 (22.5)	204 (23.2)	220 (25.1)	187 (21.3)	
College education or above	2085 (47.5)	413 (47)	404 (46.1)	437 (49.7)	413 (47.1)	418 (47.6)	
**PIR, n (%)**							0.299
< 1.3	1227 (28.0)	255 (29)	241 (27.5)	235 (26.7)	231 (26.3)	265 (30.2)	
1.3–3.5	1615 (36.8)	332 (37.8)	329 (37.6)	306 (34.8)	328 (37.4)	320 (36.4)	
≥ 3.5	1546 (35.2)	291 (33.1)	306 (34.9)	338 (38.5)	318 (36.3)	293 (33.4)	
**Smoking status, n (%)**							0.016
Never smoker	2117 (48.2)	378 (43.1)	407 (46.5)	433 (49.3)	440 (50.2)	459 (52.3)	
Former smoker	1397 (31.8)	308 (35.1)	293 (33.4)	277 (31.5)	261 (29.8)	258 (29.4)	
Current smoker	874 (19.9)	192 (21.9)	176 (20.1)	169 (19.2)	176 (20.1)	161 (18.3)	
**Drinking, n (%)**	3123 (71.2)	681 (77.6)	646 (73.7)	625 (71.1)	619 (70.6)	552 (62.9)	< 0.001
Sleep, hour	6.8 ± 1.4	6.8 ± 1.4	6.8 ± 1.5	6.8 ± 1.4	6.8 ± 1.3	6.7 ± 1.4	0.487
Physical activity (MET-hours/week)	14.0 (0.0, 56.0)	12.0 (0.0, 49.8)	14.0 (0.0, 60.0)	15.3 (0.0, 60.0)	14.0 (0.0, 56.0)	12.0 (0.0, 49.0)	0.117
Energy intake, kcal	1837.0 (1411.0, 2359.0)	1842.0 (1398.0, 2372.0)	1854.0 (1422.0, 2349.0)	1858.0 (1442.0, 2388.0)	1847.0 (1438.0, 2388.0)	1772.0 (1369.0, 2298.0)	0.105
Diabetes, n (%)	739 (16.8)	172 (19.6)	113 (12.9)	142 (16.2)	147 (16.8)	165 (18.8)	0.002
Hypertension, n (%)	2041 (46.5)	424 (48.3)	407 (46.5)	401 (45.6)	414 (47.2)	395 (45)	0.665
Phenotypic age, year	54.0 (43.9, 64.8)	56.9 (46.0, 67.3)	53.7 (44.0, 64.9)	53.6 (43.6, 64.8)	53.3 (43.2, 63.4)	52.2 (43.0, 64.0)	< 0.001
Phenotypic age acceleration, year	-1.9 (-5.7, 2.7)	-1.1 (-5.1, 4.1)	-1.8 (-5.1, 2.6)	-2.0 (-6.0, 2.2)	-2.7 (-6.2, 1.5)	-1.7 (-6.2, 3.1)	< 0.001

Continuous variables are expressed as mean ± SD or as median (interquartile range). Categorical variables are expressed as frequency (%)

*BMI* body mass index, *PIR* ratio of family income to poverty, *MET* metabolic equivalent, *CRP* C-reactive protein, *MCV* mean cell volume, *RDW* red cell distribution width, *ALP* Alkaline phosphatase, *WBC* white blood cells

### U-shaped association between serum Klotho and phenotypic age acceleration

In the multivariable linear regression analysis, the phenotypic age acceleration decreased as serum Klotho increased (per SD increment after full adjustment: β, -0.4; 95% CI, -0.65 ~ -0.15). A non-linear relationship was found between Klotho and phenotypic age acceleration when classifying Klotho into quintiles, with multivariate-adjusted β (95% CI) across the first to fifth quintiles of 2.35 (1.57 ~ 3.13), 1.17 (0.39 ~ 1.95), 0.63 (-0.15 ~ 1.41), 0, and 0.83 (0.04 ~ 1.61) (Table 2).

**Table 2:** The association between serum Klotho and phenotypic age acceleration

	n	Crude model		Model 1		Model 2		Model 3	
Serum klotho		β(95% CI))	*P* value	β(95% CI))	*P* value	β(95% CI))	*P* value	β(95% CI)	*P* value
**Continuous**									
Per SD increment	4388	-0.38 (-0.66 ~ -0.11)	0.007	-0.45 (-0.72 ~ -0.18)	0.001	-0.41 (-0.66 ~ -0.16)	0.001	-0.4 (-0.65 ~ -0.15)	0.002
**Quintiles**									
Quintile 1	878	2.74 (1.87 ~ 3.61)	< 0.001	2.59 (1.74 ~ 3.44)	< 0.001	2.36 (1.58 ~ 3.14)	< 0.001	2.35 (1.57 ~ 3.13)	< 0.001
Quintile 2	876	1.04 (0.17 ~ 1.92)	0.019	0.91 (0.06 ~ 1.76)	0.036	1.18 (0.4 ~ 1.96)	0.003	1.17 (0.39 ~ 1.95)	0.003
Quintile 3	879	0.54 (-0.33 ~ 1.41)	0.223	0.61 (-0.24 ~ 1.46)	0.16	0.63 (-0.15 ~ 1.41)	0.111	0.63 (-0.15 ~ 1.41)	0.113
Quintile 4	877	0(Ref)		0(Ref)		0(Ref)		0(Ref)	
Quintile 5	878	1.17 (0.3 ~ 2.04)	0.008	0.87 (0.02 ~ 1.72)	0.045	0.82 (0.03 ~ 1.6)	0.041	0.83 (0.04 ~ 1.61)	0.039

Model 1: adjusted for age, sex, race, smoking and drinking

Model 2: adjusted for age, sex, race, BMI, marriage, education, PIR, smoking, drinking, physical activity, energy intake, diabetes and hypertension

Model 3: adjusted for age, sex, race, BMI, marriage, education, PIR, smoking, drinking, physical activity, energy intake, diabetes, hypertension and sleep

*BMI* body mass index, *PIR* ratio of family income to poverty

**Fig. 2:**
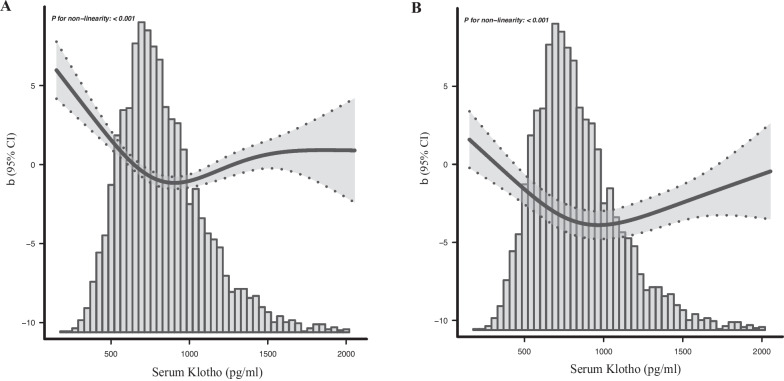
Dose–response relationship between serum Klotho and phenotypic age acceleration. (**A**: no adjustment, **B**: full adjustment)

A significant U-shaped association with phenotypic age acceleration was observed when serum Klotho was set as a continuous variable by using the smoothing curve fitting (*p* for nonlinearity < 0.001) (Fig. 2A). The U-shaped association remained significant after full adjustment (*p* for nonlinearity < 0.001) (Fig. 2B).

Accordingly, in the threshold effect analysis, the concentration of serum Klotho associated with the lowest phenotypic age acceleration was 870.7 pg/ml after full adjustment (Table 3). Phenotypic age acceleration decreased significantly with the increment of serum Klotho (per SD increment: β, -1.77; 95% CI, -2.57 ~ -0.98) in participants with serum Klotho < 870.7 pg/ml, while increased with the increment of serum Klotho (per SD increment: β, 1.03; 95% CI, 0.53 ~ 1.54) in participants with serum Klotho ≥ 870.7 pg/ml.

**Table 3:** Threshold effect analyses of serum Klotho on phenotypic age acceleration using two-piecewise regression models

Crude model			Adjusted model		
Serum klotho (pg/mL)	β(95% CI)	*P* value	Serum klotho (pg/mL)	β(95% CI)	*P* value
< 749.9	-3.50(-4.86 to -2.14)	<0.001	< 870.7	-1.77(-2.57 to -0.98)	<0.001
≥ 749.9	0.53(0.09 to 0.97)	0.019	≥ 870.7	1.03(0.53 to 1.54)	<0.001
Likelihood Ratio test		<0.001			<0.001

Adjusted for age, sex, race, BMI, marriage, education, PIR, smoking, drinking, physical activity, energy intake, diabetes, hypertension and sleep

*BMI* body mass index, *PIR* ratio of family income to poverty

### Subgroup analyses

We further performed exploratory subgroup analyses to assess the association between serum Klotho and phenotypic age acceleration in two groups of participants separated by the inflection point of serum Klotho (870.7 pg/ml).

**Fig. 3:**
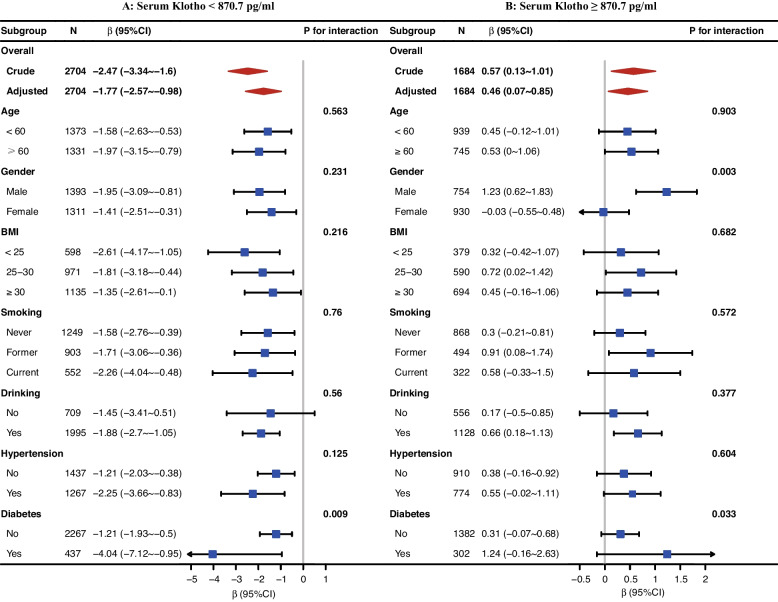
Stratified analyses for the association between serum Klotho and phenotypic age acceleration. (**A**: serum Klotho < 870.7 pg/ml, **B**: serum Klotho ≥ 870.7 pg/ml). Adjusted, if not stratified, for age, sex, race, BMI, marriage, education, PIR, smoking, drinking, physical activity, energy intake, diabetes, hypertension and sleep

None of the variables, including age, BMI, smoking, drinking, or hypertension significantly modified the association between serum Klotho and phenotypic age acceleration (all *p* for interaction > 0.05) (Fig. 3). P values for interactions for diabetes and sex among participants with serum Klotho ≥ 870.7 pg/ml; and for diabetes among participants with serum Klotho < 870.7 pg/ml, were lower than 0.05.

### Sensitivity analyses

We further performed a series of sensitivity analyses to test the robustness of the association. First, multiple imputation of missing data did not substantially change the associations (Table 4). Second, delta age estimates (i.e. ΔPhenoAge) were calculated as phenotypic age minus chronological age, and similar patterns were observed when ΔPhenoAge was used to evaluate accelerated aging (Table 5).

**Table 4:** The association between serum Klotho and phenotypic age acceleration after multiple imputation

		Crude		Adjusted model	
Serum Klotho	n	β(95% CI）	*P* value	β(95% CI）	*P* value
**Continuous**					
Per SD increment	5864	-0.3 (-0.54~-0.07)	0.012	-0.37 (-0.58~-0.15)	0.001
**Quintiles**					
Quintile 1	1172	2.3 (1.55~3.05)	<0.001	2.01 (1.34~2.68)	<0.001
Quintile 2	1173	0.54 (-0.21~1.3)	0.155	0.72 (0.04~1.38)	0.038
Quintile 3	1173	0.23 (-0.52~0.98)	0.543	0.43 (-0.24~1.1)	0.213
Quintile 4	1173	Ref		Ref	
Quintile 5	1173	0.99 (0.24~1.74)	0.01	0.72 (0.04~1.39)	0.037

Adjusted for age, sex, race, BMI, marriage, education, PIR, smoking, drinking, physical activity, energy intake, diabetes, hypertension and sleep

**Table 5:** The association between serum Klotho and ΔPhenoAge

		Crude model		Adjusted model	
Serum Klotho	n	β(95% CI)	*P* value	β(95% CI)	*P* value
**Continuous**					
Per SD increment	4388	-0.43 (-0.71~-0.16)	0.002	-0.4 (-0.65~-0.15)	0.002
**Quintiles**					
Quintile 1	878	2.84 (1.97~3.71)	<0.001	2.35 (1.57~3.13)	<0.001
Quintile 2	876	1.07 (0.2~1.94)	0.016	1.17 (0.39~1.95)	0.003
Quintile 3	879	0.56 (-0.31~1.43)	0.21	0.63 (-0.15~1.41)	0.113
Quintile 4	877	0(Ref)		0(Ref)	
Quintile 5	878	1.1 (0.23~1.98)	0.013	0.83 (0.04~1.61)	0.039

Adjusted for age, sex, race, BMI, marriage, education, PIR, smoking, drinking, physical activity, energy intake, diabetes, hypertension and sleep

*BMI* body mass index, *PIR* ratio of family income to poverty

## Discussion

In this cross-sectional study, we demonstrated a U-shaped association between serum Klotho and accelerated aging in the middle-aged and elderly US population. Serum Klotho was negatively associated with phenotypic age acceleration below the inflection point, and it was positively associated above the inflection point. Our study indicated that an optimal range for serum Klotho might exist to identify healthy aging.

The Klotho gene was identified by Kuro-O et al. in 1997 as a gene closely related to aging. Mice with a defect in the Klotho gene have a short life span and degeneration of multiple age-sensitive traits [6]. The Klotho protein, encoded by the Klotho gene, is a transmembrane protein that acts as a co-receptor for fibroblast growth factor (FGF)-23. Ectodomain shedding of the extracellular domain produces the secreted form of Klotho, which can be detected in serum, urine, and cerebrospinal fluid [14]. Serum Klotho declines physiologically after 40 years old and is easy to measure [15].

In recent years, Klotho has emerged as a powerful regulator of the aging process by regulating many pathways [8, 16, 17]. It is regarded as a crucial role in the pathophysiology of common aging-related disorders, including chronic kidney disease, metabolic syndrome, and cardiovascular diseases [7, 9, 18]. In addition, reduced serum Klotho has been reported to be significantly associated with an increased risk of death [19]. However, most previous studies have shown a linear inverse association between Klotho and the risk of age-related disease and associated mortality. Emerging evidence suggests that high levels of Klotho are not always associated with improved health [20]. Recently, Chen et al. have found that Klotho had a U-shaped relationship with mortality among people with diabetes [21]. A case-cohort analysis conducted in Canada also exhibits the association of Klotho with fracture followed a U-curve [22]. Here we found a U-shaped association between serum Klotho levels and phenotypic age acceleration, with optimal concentrations of serum Klotho associated with the lowest phenotypic age acceleration. The results suggest that keeping Klotho in an appropriate range might be conducive to promoting healthy aging.

The mechanism underlying the U-shaped association between serum Klotho levels and accelerated aging remains unknown. Klotho plays the anti-aging role by regulating many pathways that contribute to aging, such as phosphate homeostasis, oxidative stress, insulin/insulin-like growth factor 1 signaling, and Wnt signaling [17]. Moreover, Klotho may be involved in the negative regulation of vitamin D by regulating 1α-hydroxylase expression [23]. Klotho^−/−^ mice show a high vitamin D activity and exhibit features of premature aging, which can be reversed by normalizing vitamin D [24], implying the premature-aging-like features might be due to hypervitaminosis D. Conversely, overexpression of Klotho leads to vitamin D deficiency [21], which has also been reported to promote aging [25–28]. The U-shaped response curve of aging and vitamin D status might explain the association between serum Klotho and accelerated aging in the present study. More research into the underlying mechanisms is recommended. As Klotho administration has been regarded as a promising method to improve health, possible optimal ranges or therapeutic windows should be considered in studies targeting Klotho enhancement [13].

Although interaction between diabetes and Klotho (≥ 870.7 pg/ml), sex and Klotho (≥ 870.7 pg/ml), as well as diabetes and Klotho (< 870.7 pg/ml) on accelerated aging was observed in the present study, due to chance given multiple testing and similar directionality of most of the associations, these results may not have significant clinical implications. Moreover, the different outcomes in diabetes and non-diabetes groups might be partially due to the skewed sample size. Future studies of a larger sample size are needed to replicate these observed interactions.

This is the first to evaluate the dose–response relationship between serum Klotho and accelerated aging. However, there are also some limitations. First, considering the cross-sectional nature of the NHANES data, the establishment of causality was precluded. Second, serum Klotho in the NHANES database was only tested in participants aged 40–79 years old and the role of serum Klotho in youngers was inconclusive. Third, although we adjusted for as many confounders as possible, the possibility of unmeasured confounders cannot be ruled out. Finally, phenotypic age calculated by using chronological age and 9 aging-related biomarkers was employed to assess aging in the present study, considering data availability in NHANES database. A number of aging measures have been proposed in previous studies, the most prominent being epigenetic clocks based on measures of DNA methylation [29]. Given that different aging metrics capture aging performance at various level in estimating adverse health outcomes, it is necessary to evaluate the association of Klotho with other aging measures in future studies, including other longitudinal signatures of aging [30].

## Conclusion

We found a U-shaped association of serum Klotho with accelerated aging in the middle-aged and elderly US population. Further studies should be performed to confirm these findings and explore the underlying mechanisms.

## Data Availability

The data sets generated and/or analyzed during the current study are available in the NHANES database repository: https://wwwn.cdc.gov/nchs/nhanes/continuousnhanes/default.aspx?BeginYear=2007 and https://wwwn.cdc.gov/nchs/nhanes/continuousnhanes/default.aspx?BeginYear=2009.

## Abbreviations

NHANES: National Health and Nutrition Examination Survey
CRP: C-reactive protein
BMI: Body mass index;
PIR: Poverty income ratio
MET: Metabolic equivalent
CI: Confidence intervals
SD: Standard deviations
IQR: Interquartile range
